# Awareness Campaigns in a Horizontally Differentiated Market with Environmentally Conscious Consumers, Private Versus Public Duopoly

**DOI:** 10.3390/ijerph191912891

**Published:** 2022-10-08

**Authors:** Hamid Hamoudi, Carmen Avilés-Palacios

**Affiliations:** 1 Departamento de Fundamentos de Análisis Económico, Universidad Rey Juan Carlos-URJC, Paseo Artilleros s/n, 28032 Madrid, Spain; 2 ETSI Montes, Forestal y del Medio Natural—Universidad Politécnica de Madrid-UPM, Calle José Antonio Novais 10, 28040 Madrid, Spain

**Keywords:** differentiated duopoly, environmental regulation, sustainability, awareness campaigns, consumer awareness

## Abstract

This paper examines horizontally differentiated duopolies à la Hotelling with environmentally conscious consumers and a planner promoting a sustainable good with costly awareness campaigns (ACs). The objective is to find the planner’s optimal strategies and their effects on the firms’ behaviour. The analysis is carried out with two approaches, considering a private and a public duopoly. In both, it is shown that the planner chooses the average characteristic supported by a higher intensity campaign. However, with the private one, such an outcome is possible if the planner has minimal resources. Consumer consciousness and ACs have opposite effects on the firms and the planner. It is proven that consumer awareness favours the interests of the duopolies and reduces those of the planner, while the contrary is true for ACs. Finally, it is shown that a public duopoly is the best scenario for sustainability. This study provides an environmental policy to replace or complement traditional instruments and a more suitable business framework to achieve efficient results.

## 1. Introduction

Reducing the negative impacts of human activities on the environment increasingly requires government intervention. To promote this development, the United Nations continuously calls for a review of the consumption and production models of industrialized states in accordance with the Paris Agreement and the Sustainable Development Goals [1]. In terms of consumption and production patterns, we highlight two government policies, sanctioning and preventive policies. Sanctioning policies, however, are often unpopular from a business perspective and not so popular from a consumer point of view. Taxing unsustainable options increases their price, and price is an important factor for many when purchasing products and services. Moreover, from the public planner’s perspective it is difficult to discern who, when, and how much to pay (for example two firms polluting the same river). Preventive policies avoid these problems and shift the responsibility burden to polluters in a direct and transparent way. A widely used policy tool to prevent environmental damage are awareness campaigns (AC) aimed at generating and developing an environmental consciousness among consumers. As an example of an institutional AC, the European Commission started the campaign “You Control Climate Change” in 2006 with the aim of informing people about environmental damage, initiating proactive dialogues and targeting (small) behavioural changes [2]. To achieve this goal, the EU has heavily invested in tools such as advertising, websites, exhibitions, media relations, events, and school programs, both at the European and national level. In addition, the EU has financed national awareness-raising campaigns in its Member States. In the 1990s, the EU public authorities decided to dedicate resources to ACs to dissuade consumers from buying spray deodorants [3]. This type of product was considered highly polluting due to CO2 emissions. The campaigns were so effective that the demand was quickly reduced. The product stopped being offered and was substituted by roll-on deodorants. Nowadays, both products coexist but spray deodorants have been technically improved to achieve zero emissions, whereas roll-on deodorants have taken most of the market share. In the last decade, many government campaigns have been carried out to inform citizens about the negative externalities of bad consumption habits. These campaigns focus more specifically on sectors such as energy, automotive, and food. Other non-governmental organizations, such as environmental groups (e.g., Ecologists in Action, Friends of the Earth, Greenpeace, and World Rainforest Movement) have also run even more persuasive and very aggressive ACs.

Specifically, ACs have been proven effective in increasing consumer involvement in environmental issues, modifying their consumption preferences, and avoiding environmentally unfriendly products. Some defenders, based on Schwartz’s theory of moral motivation of norms [4,5] and reviewed by Turaga et al. [6], proclaim that “inducing environmental behaviours in individuals is one of the most important challenges on the road to sustainability”. For example, Van der Made and Schoonbeek consider that persuasive awareness-raising operations aggravate consumers’ environmental concerns [7]. Sartzetakis et al. examine, in a dynamic framework, the role of information on environmental damage related to the consumption of certain products as a policy instrument complementing environmental taxes [8]. In their model, an advertising campaign helps to reduce the information asymmetry between the population and the business world. Lambertini carefully examines the possibility that environmentally conscious consumers can regulate corporate behaviour even in the absence of an explicit policy measure [9]. Kaufman proposes a dynamic learning model to investigate whether financial incentives or informative advertising campaigns are more effective in encouraging green purchases [10]. Moreover, several studies considering product differentiation models have assumed that consumer environmental awareness (CEA) affects consumption preferences. Some authors applying horizontal product differentiation based on the Hotelling model [11] study the case in which environmentally conscious consumers can regulate the behaviour of firms in the absence of public intervention [12,13,14]. On the other hand, other authors, using vertical differentiation models, have analysed governmental or environmental groups’ strategies that consider environmental ACs [7,15,16].

This study investigates the effects of sensibilization campaigns on the decisions of firms in terms of their product and price choices. In Hotelling’s spatial duopoly an environmental planner proposes a sustainable production characteristic that he promotes through an AC. The benchmark sustainability characteristic does not a priori mean that it is the most sustainable. The success of public policy is to offer healthy and sustainable choices by default along with the freedom not to use them [17]. For instance, one may consider the promotion of electric cars, which are also known to be polluting and not as environmentally friendly as claimed. To carry out the promotion, the planner designs an AC to inform consumers that the consumption of a characteristic different from the reference characteristic generates external harm. The level of the campaign depends on the budget the planner allocates to it. It is assumed that there is a direct effect of the AC on consumers that will be further reflected in their preferences due to a bad conscience. Against this background, consumers adjust purchases taking into account the effect of their personal awareness and the effect of the AC. The consumer preference structure depends simultaneously on price, the effect of personal awareness, and the effect of ACs. The key point here is that ACs become a useful policy instrument, which allows us to define them as an “indirect regulatory” tool. This is not just a theoretical construction but a tool to deal with several real-life competition situations where environmental issues turn into a strategic variable (i.e., spray vs. roll-on deodorant example). On the other hand, the choice of the reference characteristic, as well as the choice of the budget, are determined by criteria defined by the planner himself. The model is established and analysed first with a private duopoly and then with a public duopoly.

The structure of the paper is the following: Section 2 provides a literature review. Section 3 describes the model. Section 4 analyses the market with a private duopoly, and it is divided into three subsections: Section 4.1 and Section 4.2, where equilibrium in prices and characteristics is found, respectively, whereas Section 4.3, calculates the optimal level of the AC and the optimal sustainable characteristic of the environmental planner. Section 5 examines the proposed model with a public duopoly. In Section 5.1, the public manager defines the optimal characteristics of the firms, and in Section 5.2, the environmental planner determines the optimal level of the AC and sustainable characteristics. Section 6 provides a comparative analysis of the two proposals, and conclusions are presented in Section 7.

## 2. Related Literature

This work is framed within the theoretical literature on environmental policies related to product differentiation. Several authors have considered regulatory instruments based on fees or subsidies, and/or the imposition of a product standard, and/or an environmentally conscious consumer policy. Others have analysed the interaction between environmental policies and the environmental behaviour of firms or consumers using different approaches. Indicatively, Marini et al. [18], considering a differentiated duopoly, study how the supply side of greening affects the way firms choose their prices and products and the resulting consequences for the overall level of pollution. They find that environmentalism does not necessarily lead to a better environmental outcome, as it gives green firms greater market power which they use to charge higher prices. However, it can be used to effectively complement more traditional policy instruments, such as a minimum environmental standard. Ambec and De Donder [19] analyse an economy with two types of citizens, named neutral and green, who consume a whole unit of a polluting good and where green firms differentiate products according to their environmental quality. They contrast two ways of public intervention: (1) an environmental quality standard and, (2) a pollution tax. They consider an arbitrary pollution target. For any given level of pollution, emission taxes turn out to be less cost-effective than an emission standard because taxes always induce a higher wedge between the environmental qualities of products. On the contrary, consumers prefer taxes to standards when the intensity of the warm glow is not too great. Arguedas and Rousseau [20], analyse the behaviour of a monopolist and a duopolistic system with four different approaches: (1) an initial laissez-faire approach; (2) a policy of raising the environmental awareness of consumers; (3) the imposition of a product standard; and (4) the application of a technology subsidy. They conclude that a policy based on consumer education will induce both the monopolist and duopolistic system to increase the energy efficiency of the product and to charge a higher price. As for the imposition of a product standard, they find that such a policy can counteract the negative effects of displacement of consumers’ intrinsic motivation in a monopoly environment, although this counteracting effect is less powerful under a duopoly. However, a subsidy does not provide such a support system and the full effect of exclusion will be visible. Karakosta [21] examines the effects of tax competition on environmental product quality, pollution, and well-being in a two-country duopoly where consumers are environmentally conscious. Hsu et al. [22] show the implications of consumer awareness on environmental policy in both mixed and private oligopolies under regulated entry and free entry. Although these studies use different models of vertical differentiation with different objectives from each other, they all highlight in one way or another the effect of consumer environmental awareness (CEA) on the market.

As described in the previous section, our work relates horizontal differentiation models by attributing environmental awareness to consumers. The main difference is that our scenario models CEA to depend on regulatory policy and furthermore considers the interaction between the regulator and firms. However, related studies either assume the two independent variables to be independent of each other or divide consumers into groups. Among others, Eriksson [12] and Conrad [13], analyse price competition and product differentiation with green consumers. However, they do not endogenise environmental regulation with a political economy approach. Clemenz [23] investigates the impact of eco-labels on the reduction of emissions in a market with horizontal differentiation à la Hotelling [11] and à la Salop [24]. He finds that the reduction method makes a difference in the effectiveness and efficiency of eco-labels. Espínola-Arredondo and Zhao [25], analyse Hotelling’s linear city model [11] with two types of consumers, green and brown, where the final products of the two firms are symmetric except for their environmental impact. In their efficiency comparison, they find that environmental regulation produces higher social welfare than no policy. Mantovani and Vergari [16], using the two differentiation models (vertical and horizontal), compare two policy instruments that can be adopted to curb carbon emissions. The first is a conventional pollution tax, the second is an environmental campaign that raises consumers’ awareness of the relative impact of their consumption choices. They show that the relative effectiveness of the two policy instruments depends critically on consumers’ initial concern for the environment.

Much closer to our model, He and Deng [14], in a model of horizontal differentiation à la Hotelling [11], introduce CEA which they divide into two main elements: (1) a subjective element and, (2) a social element. They analyse how price and competitor characteristics are affected by the two elements of environmental awareness. There are, nevertheless, some essential differences linked to our contribution to the literature. This paper, in contrast to previous studies, and especially to He and Deng [14], introduces an environmental authority as the agent implementing a policy that incentivizes the promotion of sustainable goods. Therefore, the second component of environmental awareness is related to awareness campaigns and not to the average characteristic of the total consumer population. Consequently, utility functions for consumers share a similar structure but are notionally different. The number of players and the timing used to formalize the interaction games amongst players constitutes another essential difference with He and Deng [14].

The analysis is carried out firstly with a private duopoly and, secondly, with a public duopoly, and it is based on multi-stage non-cooperative games. Considering a private duopoly, the model is formulated as a three-stage game. Firstly, the environmental planner simultaneously chooses the benchmark characteristic and the budget of the AC, then the firms compete on the characteristics of the goods and then on prices. With the public duopoly, the formalisation is given by a two-stage game: the environmental planner simultaneously chooses the benchmark characteristic and the budget of the AC, and then the public manager of both firms decides on the production sustainability characteristics of each. In both scenarios, the backward induction methodology is used. Finally, a comparative analysis of the standard model of horizontally differentiated products, with and without environmental regulation, is carried out.

Although the literature reported above is not exhaustive, we are not aware of any work that considers a distinct market for an environmental authority that proposes two strategic variables, a reference environmental characteristic and a budget for its promotion. This study contributes to the literature on environmental economics and industrial management by providing a framework for determining the environmental characteristic of a product, the level of AC that should support it, and the characterization of an appropriate industrial environment.

## 3. The Model

A horizontally differentiated market is considered, in which goods are represented by their sustainability, and an environmental planner proposes a certain sustainability benchmark that it promotes using an AC. This campaign is supposed to help develop “bad conscience” in the consumer by emphasising the environmental damage caused by the consumption of goods with different characteristics to those proposed by the planner. In other words, pro-environmental behaviour is stimulated by encouraging the individual to want to consume sustainable goods, as considered by the environmental planner. This preventive approach to environmental protection can indirectly lead firms to reduce polluting products.

The framework of this contribution builds on the standard Hotelling’s model described by D’Aspremont et al. [26]. It is assumed that the market is composed of products ranked, in increasing order, by their level of sustainability within a [0, 1] spectrum, where 0 represents the lowest level of sustainability and 1 the highest. There are two firms producing the same good that only differ in their sustainability characteristics denoted by $x_{1},\;x_{2}$, in [0,1], such that $x_{1}\leq \;x_{2}$. The firms can be privately or publicly managed. In the first case, each firm is managed independently and in the second case, business decisions are taken by the same public manager. There is a continuum of consumers uniformly distributed in the interval [0, 1] and represented by their sustainability characteristics denoted by, x. Each consumer buys a single unit of the product, $x_{i}$, at price $p_{i},\;i=1,2$. In addition to the price, when a consumer acquires the product with sustainability characteristic, $x_{i}$, different from their preferred characteristic, x, they incur a cost represented by

(1) $$T\left(x,x_{i},t\right)=t{\left(x-x_{i}\right)}^{2},\;i=1,\;2.$$

Parameter t>0 measures the degree of environmental sensitivity of consumers. The cost function $T\left(x,x_{i},t\right)$ can be regarded as the loss of utility that a consumer would incur due to their environmental sensitivity. Furthermore, consumers are informed about the environmental impact of the goods they buy through environmental sensitivity campaigns conducted by a planner. The campaigns are articulated around two strategic variables:

1. A sustainability benchmark θ∈[0, 1] which represents the planner’s valuation of environmental quality. The variable θ is not a standard imposed on firms, but rather a public promotional information. The sustainability characteristic θ in our paper is different from that in He and Deng [14]. They set θ as the average level of environmental friendliness of all the sold products
2. A budget $\gamma \in \left[0,\bar{\gamma }\right]$ to promote the planner’s characteristic  θ, where $\bar{\gamma }$ is a positive real number that represents the maximum budget available to the environmental planner; $\bar{\gamma }$ is set by the government.

Combining these two variables, the planner designs an AC to promote consumers’ environmental sensitivity by advising that the consumption of products with a characteristic $x_{i}$ different from characteristic θ generates external damage. Thus, in this model, environmental damage is not related to polluting emissions as in most studies; rather it is associated with the deviation from the sustainability benchmark in consumption. This research considers the environmental damage scheme as a quadratic function of the deviation from the “promoted sustainability characteristic” whose expression is

(2) $$d\left(x_{i},\theta \right)={\left(\theta -x_{i}\right)}^{2}\;,\;\;\;i=1,\;2.\;$$

Defined as the damage function $d\left(x_{i},\theta \right)$, the planner uses its budget γ as a catalyst for the damage in order to raise consumers’ awareness through advertisements. Therefore, the AC is described as a function of the budget and the damage, and formalised as

(3) $$L\left(x_{i},\theta ,\;\gamma \;\right)=\gamma \;d\left(x_{i},\theta \right)\;\;i=1,\;2.$$

The AC is a tool to stimulate environmental awareness, in a similar way to advertisements, against unhealthy habits, such as smoking (with campaigns showing the damage caused by nicotine on the respiratory system) or reckless driving (showing the human losses caused by inappropriate behaviour on the road). A higher (lower) budget (γ) implies a higher (lower) level of the environmental sensitivity campaign.

The consumers internalise the campaign message in their preferences in terms of remorse feelings. For simplicity, we assume that all consumers are affected in the same way. Therefore, the campaign is included as an additional loss in the consumer’s utility, and its expression is given by

(4) $$u\left(x,x_{i}\right)=k-p_{i}-T\left(x,x_{i},t\right)-L\left(x_{i},\theta ,\gamma \right),\;i=1,2.\;$$

where k indicates the consumer surplus and it is assumed to be large enough that all consumers buy the product. Compared to the standard model of horizontal differentiation, the utility of purchasing the good from firm i=1,2 is modified by introducing the term $\;L\left(x_{i},\theta ,\gamma \right)$. It follows that utility may be diminished by the purchase of a product different to the planner’s characteristic  θ. As mentioned above, the larger the budget value γ assigned to the campaign, the larger the campaign’s impact on consumers. On the contrary, when  γ→0, the standard model is restored

The indifferent consumer is obtained by considering that utility functions are equal when buying from any of the two firms. The sustainability characteristic denoted by $\hat{x}$ for the indifferent consumer can be written as follows

(5) $$\hat{x}=\frac{p_{2}-p_{1}}{2t\left(x_{2}-x_{1}\right)}+\frac{1}{2}\left(x_{2}+x_{1}\right)+\frac{\gamma }{2t}\;\left(x_{2}+x_{1}-2\theta \right).\;$$

As expected, the characteristic $\hat{x}$ of the indifferent consumer depends not only on the sustainability characteristics and prices of firms, and the degree of environmental sensitivity of consumers, t, but also on the environmental policy instruments (θ,γ). In expression (5), the first two terms represent the solution of the standard model. The last term represents the effects of environmental regulation.

Since consumers  x with $x<\hat{x}$ buy from firm 1, while consumers  x with $x>\hat{x}$ choose firm 2, demand functions are written as

(6) $$\;Q_{1}=\hat{x},\;\;Q_{2}=1-\hat{x}.\;$$

The incorporation of AC affects demand, which will lead firms to adjust their strategies in prices and products to the new scenario. Without loss of generality, production costs are assumed to equal zero, due to the fact they will not substantially affect the results, according to the calculations performed and which are made available to the reader on request, but on the other hand, they do increase the complexity of the model in terms of interpretation) and the profit function of each firm is

(7) $$B_{i}=p_{i}Q_{i},\;i=1,2$$

When the firms have the same public manager, the objective function is social welfare  W, usually defined as the sum of consumers’ surplus ${\;E}_{C}$ and producers’ surplus $E_{P}$, whose expressions are

(8) $$W=\int_{0}^{\hat{x}}u\left(x,x_{i}\right)dx+\int_{\hat{x}}^{1}u\left(x,x_{i}\right)dx+\overset{2}{\underset{i=1}{\sum }}B_{i}$$

Regarding environmental regulation policy, a mechanism based on awareness-raising campaigns is proposed as an alternative to conventional instruments via taxes or penalties. The planner assumes that the higher the environmental sensitivity of consumers, the lower the deviation in consumption, and therefore the lower the environmental damage. To reflect this assumption, a mechanism is designed to represent the total damage per monetary unit spent on awareness-raising campaigns, the formal expression of which is

(9) $$\Phi \;\left(\theta ,\gamma \;\right)=\frac{D\left(x_{1},x_{2},\theta \;\right)}{\gamma }.\;$$

where $D\left(x_{1},x_{2},\theta \;\right)$ represents the global environmental damage as follows

(10) $$D\left(x_{1},x_{2},\theta \;\right)=\int_{0}^{\hat{x}}d\left(x_{1},\theta \right)\;dx+\int_{\hat{x}}^{1}d\left(x_{2},\theta \right)dx.\;\;$$

The objective is to determine the sustainable characteristic θ, and the budget γ, that minimise the unit damage function Φ(θ,γ ). Therefore, the problem of the planner is

(11) $$\underset{\;\theta ,\;\gamma }{\;Min}\;\Phi \left(\theta ,\gamma \;\right).\;$$

As mentioned above, the model considers two scenarios: firstly, a game theoretical framework with a private duopoly, and secondly with a public duopoly. The timing of each scenario is as follows

1. The first approach develops as a game in three stages. In the initial stage, the planner chooses simultaneously characteristic  θ and the budget  γ, in the second one, the firms set their production characteristics $\;\left(x_{1},x_{2}\right)$; and in the final stage, they choose the prices $\;\left(p_{1},p_{2}\right)$.
2. The second approach considers a game in two stages. First, the environmental planner chooses simultaneously the sustainability benchmark and the budget, (θ,γ) ; and then, the public manager of the two firms sets the characteristics $\;\left(x_{1},x_{2}\right)$.

It is assumed that the planner simultaneously sets the sustainability benchmark and the budget in anticipation of the reaction of the firms or the public manager in terms of sustainability levels and prices. In both cases, the solutions are obtained by backward induction.

## 4. Optimal Strategies from a Business Perspective—Private Duopoly

Strategic interaction is analysed in this section as a sequential game between the planner and the firms. The section is organised as follows: Section 4.1 establishes equilibrium in prices; Section 4.2 determines the optimal sustainability characteristics once optimal pricing strategies are set; Section 4.3 concludes analysis of the Nash subgame analysis by finding the optimal budget and sustainable production characteristics.

### 4.1. Price Equilibrium

At this game stage, firms compete in prices. Assuming the rival’s price is fixed, each firm maximizes its profit with respect to its price given the sustainability characteristic (θ) and budget (γ) set by the planner and the product characteristics $\;\left(x_{1},x_{2}\right)$. Then, considering the profit function of each firm given in Equation (7), the price equilibrium is derived from first-order conditions as follows:

(12) $$\;p_{1}^{E}=\frac{\left(x_{2}-x_{1}\right)}{3}\left[t\;\left(2+x_{2}+x_{1}\right)+\gamma \left(x_{2}+x_{1}-2\theta \right)\right],\;$$

(13) $$\;p_{2}^{E}=\frac{\left(x_{2}-x_{1}\right)}{3}\left[t\;\left(4-x_{2}-x_{1}\right)-\gamma \left(x_{2}+x_{1}-2\theta \right)\right].$$

Considering that $0\leq \;\hat{x}\leq 1$, $\left(p_{1}^{E},p_{2}^{E}\right)$ will be an equilibrium price if and only if:

(14) $$\frac{2\left(\;\gamma \theta -t\right)}{\left(t+\gamma \right)}\leq \left(x_{2}+x_{1}\right)\leq \frac{2\left(\;2t+\gamma \theta \right)}{\left(t+\gamma \right)}.$$

Substituting $p_{1}^{E}$ and $p_{1}^{E}$ into the the indifferent consumer $\hat{x}$, the firms’ market shares are formulated as follows:

(15) $$\;Q_{1}^{E}=\frac{1}{6}\left(x_{2}+x_{1}+2\right)+\frac{\gamma }{6\;t}\;\left(x_{2}+x_{1}-2\theta \right),$$

(16) $$\;Q_{2}^{E}=\frac{1}{6}\left(4-x_{2}-x_{1}\right)-\frac{\gamma }{6\;t}\;\left(x_{2}+x_{1}-2\theta \right).\;$$

Next, the optimal sustainability characteristics are determined.

### 4.2. Sustainability Characteristics Equilibrium

To examine the competition in sustainability levels, the profits at the price equilibrium are considered. Their expressions are obtained by substituting respectively Equations (12), (13), (15), and (16) into Equation (7), and are summarized below

(17) $$\;B_{1}^{E}\left(x_{1},x_{2}\right)=\frac{\left(x_{2}-x_{1}\right)}{18t}\;{\left\{t\left[2+\left(x_{2}+x_{1}\right)\right]+\gamma \left[\left(x_{2}+x_{1}\right)-2\theta )\right]\right\}}^{2}\;$$

(18) $$\;B_{2}^{E}\left(x_{1},x_{2}\right)=\frac{\left(x_{2}-x_{1}\right)}{18t}\;{\left\{t\left[4-\left(x_{2}+x_{1}\right)\right]-\gamma \left[\left(x_{2}+x_{1}\right)-2\theta )\right]\right\}}^{2}.\;$$

The optimal sustainability characteristic of each firm is determined by maximizing Equations (17) and (18) respectively; thus, based on the first-order conditions, the solution is

(19) $$\;x_{1}^{*}=\frac{\left(4\gamma \theta -t\right)}{4\left(t+\gamma \right)}\;,\;\;x_{2}^{*}=\frac{\left(4\gamma \theta +5t\right)}{4\left(t+\gamma \right)}$$

The characteristics $\;\left(x_{1}^{E},x_{2}^{E}\right)$ will be in equilibrium when, simultaneously,$\;x_{1}^{E}$ maximizes $\;B_{1}^{E}\left(x_{1},x_{2}^{E}\right)$ in  [0,1], and $\;x_{2}^{E}$ maximizes $\;B_{2}^{E}\left(x_{1}^{E},x_{2}\right)$ in  [0,1]. Therefore, the above result $\left(x_{1}^{*},x_{2}^{*}\right)$ would not always represent an equilibrium (for γ=0 and/or θ=0 (unregulated market), when firms can be located in the interval (−∞,+∞), the solution is given $x_{1}^{*}=\left(-1/4\right)$, $x_{2}^{*}=\left(5/4\right)$ (see [27,28]); however, if firms can be located only in  [0,1], the solution follows $x_{1}^{*}=0$, $x_{2}^{*}=1$ (see [26])) because they do not belong to  [0,1] for any  t, θ and  γ. From the Equation (19), several cases, in which equilibrium can be reached, are analysed. Given that for any positive t, θ, and  γ, $x_{1}^{*}\leq 1$ and $0\leq x_{2}^{*}$ are always satisfied, the other alternatives to consider for the equilibrium calculation are as follows:

i. First case: $x_{1}^{*}\leq 0,\;x_{2}^{*}\geq 1$ is equivalent to $\gamma \leq Min\left\{\frac{t}{4\left(1-\theta \right)},\frac{t}{4\theta }\right\}$.
ii. Second case: presents two alternatives:
  - $x_{1}^{*}\leq 0,\;0\leq x_{2}^{*}\leq 1$ is equivalent to $\frac{t}{4\left(1-\theta \right)}\leq \gamma \leq \frac{t}{4\theta }$, if $\theta \in \left[0,\frac{1}{2}\right]$
  - $0\leq x_{1}^{*}\leq 1,\;x_{2}^{*}\geq 1$ is equivalent to $\frac{t}{4\theta }\leq \gamma \leq \frac{t}{4\left(1-\theta \right)}$, if $\theta \in \left[\frac{1}{2},1\right]$
iii. Third case: $\left(0\leq x_{1}^{*}\leq 1\right),\;\left(0\leq x_{2}^{*}\leq 1\right)$ is equivalent to $Max\left\{\frac{t}{4\left(1-\theta \right)},\frac{t}{4\theta }\right\}\leq \gamma$.

These three cases correspond to different levels of the budget γ allocated to the AC. To distinguish the impact of each level on the equilibrium, each case is studied separately in the following sections.

#### 4.2.1. First Result: Corner Solution

Considering the first case (i) in which the level of the AC is assumed to be relatively small, the result corresponds to a corner solution, and it is given in the following proposition.

**Proposition** **1.** *For any*θ∈[0,1], t>0,*and*$\gamma \leq Min\left\{\frac{t}{4\left(1-\theta \right)},\frac{t}{4\theta },\;\bar{\gamma }\right\}$, *the characteristics equilibrium is*:

(20) $$x_{1}^{E1}=0,\;x_{2}^{E1}=1.\;$$

**Proof.**  For θ ∈[0,1] and $\gamma \leq Min\left\{\frac{t}{4\left(1-\theta \right)},\frac{t}{4\theta },\;\bar{\gamma }\right\}$, with $\left(x_{1}^{*},\;x_{2}^{*}\right)$ given by the expression (19) it is verified that ($x_{1}^{*}\leq 0,\;x_{2}^{*}\geq 1$ ). Thus $Arg\underset{x_{1}\in \left[0,1\right]}{Max}\;B_{1}^{E}\left(x_{1},1\right)=0,$ and $\underset{x_{2}\in \left[0,1\right]}{Max}\;B_{2}^{E}\left(0,x_{2}\right)=1.$ So the equilibrium is (0,1). □

The firms choose the extreme sustainability characteristics, which correspond to maximum differentiation. Firm 1 chooses the least possible level of sustainability and firm 2 the maximum. This is due to the limited budgetary capacity of the planner. The planner’s strategies (θ,γ) do not have enough impact to influence the decisions of the firms in terms of sustainability levels, but they do influence prices and demands, as can be seen through the following expressions:

(21) $$p_{1}^{E1}=t+\frac{\gamma \left(1-2\theta \right)}{3},p_{2}^{E1}=t-\frac{\gamma \left(1-2\theta \right)}{3},\;$$

(22) $$Q_{1}^{E1}=\frac{1}{2}+\frac{\gamma \left(1-2\theta \right)}{3},Q_{2}^{E1}=\frac{1}{2}-\frac{\gamma \left(1-2\theta \right)}{3}\;.\;$$

In this case, price and demand for firm 1 are increasing with respect to  (γ), while price and demand of firm 2 are decreasing. Now, if θ=(1/2), price equilibrium remains unaffected, thus replicating the unregulated market results.

#### 4.2.2. Second Result: Hybrid Solution

Characteristics equilibrium is now analysed under the second case, given by (ii), which corresponds to a moderate level of AC.

**Proposition** **2.** *For any*,  t>0, *and*$\gamma \;\in \left[0,\;\bar{\gamma }\right]$*the characteristics equilibrium is*:

$$\left(a\right)\;x_{1}^{E2}=0,\;x_{2}^{E2}=x_{2}^{**}\;if\;and\;only\;if\;\theta \in \left[0,\frac{1}{2}\right]\;and\;\frac{t}{4\left(1-\theta \right)}\leq \gamma \leq \;Min\left\{\frac{t}{4\theta },\;\bar{\gamma }\right\}$$

$$\left(b\right)\;x_{1}^{E2}=x_{1}^{**},\;x_{2}^{E2}=1\;if\;and\;only\;if\;\theta \in \left[\frac{1}{2},1\right]\;and\;\frac{t}{4\theta }\leq \gamma \leq \;Min\left\{\frac{t}{4\left(1-\theta \right)},\;\bar{\gamma }\right\}$$

*where*:

(23) $$\;x_{1}^{**}=\frac{\gamma \left(2\theta +1\right)-t}{3\left(t+\gamma \right)}\;,\;\;x_{2}^{**}=\frac{2\left(2t+\gamma \theta \right)}{3\left(t+\gamma \right)}.\;\;$$

**Proof.**  Given $\theta \;\in \left[0,\frac{1}{2}\right]$ and $\frac{t}{4\left(1-\theta \right)}\leq \gamma <Min\left\{\frac{t}{4\;\theta },\;\bar{\gamma }\right\}$, for any $\;x_{1}\in \left[0,1\right];\;B_{1}^{E}\left(x_{1},x_{2}\right)$ reaches a maximum in $x_{1}^{E2}=0$. In this case, the best response of firm 2 is $x_{2}^{**}$ given by Equation (23) which belongs to [0,1] and satisfies the second-order condition. Thus, the equilibrium is $\;\left(x_{1}^{E2}=0,\;x_{2}^{E2}=x_{2}^{**}\right)$.

Given $\theta \;\in \left[\frac{1}{2},\;1\right]$ and $\frac{t}{4\theta }\leq \gamma \leq \;Min\left\{\frac{t}{4\left(1-\theta \right)},\;\bar{\gamma }\right\}$, for any $\;x_{2}\in \left[0,1\right];\;B_{2}^{E}\left(x_{1},x_{2}\right)$ reaches a maximum in $x_{2}^{E2}=1$. In this case, the best response of firm 2 is $x_{1}^{**}$ given by Equation (23) which belongs to [0,1] and satisfies the second-order condition. The equilibrium is $\;\left(x_{1}^{E2}=x_{1}^{**},\;x_{2}^{E2}=1\right)$. □

With a slightly higher budget γ than in the first case, the planner forces one of the firms to change its strategy, and this change leads to a reduction in product differentiation and more intense price competition. Prices and demand are given by:

Case (a):

(24) $$\;p_{2}^{E1}=\frac{4\left(2t+\gamma \theta \right)\left(5t-2\gamma \theta \right)}{27\left(t+\gamma \right)},\;\;p_{2}^{E1}=\frac{8{\left(2t+\gamma \theta \right)}^{2}}{27\left(t+\gamma \right)},\;\;$$

(25) $$\;Q_{1}^{E1}=\frac{1}{2}+\frac{\left(t-4\gamma \theta \right)}{18t},\;\;Q_{2}^{E1}=\frac{1}{2}-\frac{\left(t-4\gamma \theta \right)}{18t}.\;\;$$

Case (b):

(26) $$\;p_{2}^{E1}=\frac{8{\left(2t+\gamma \left(1-\theta \right)\right)}^{2}}{27\left(t+\gamma \right)},\;p_{2}^{E1}=\frac{4(2t+\gamma \left(1-\theta \right)\left(5t-2\gamma \left(1-\theta \right)\right)}{27\left(t+\gamma \right)}\;,\;\;$$

(27) $$Q_{1}^{E1}=\frac{1}{2}-\frac{\left(t-4\gamma \left(1-\theta \right)\right)}{18t},\;\;Q_{2}^{E1}=\frac{1}{2}+\frac{\left(t-4\gamma (1-\theta \right))}{18t}.\;\;$$

In both cases, one of the firms has a competitive advantage in terms of price, demand, and profit. Here, also the uniqueness of the equilibrium depends on the upper limit of $\bar{\gamma }$

(28) $$\bar{\gamma }\;\geq Max\left\{\frac{t}{4\theta },\frac{t}{4\left(1-\theta \right)}\;\right\}.\;\;$$

In any case, if θ=(1/2) and $\gamma =\bar{\gamma }=\left(t/2\right)$, the result of Proposition 1 is restored. Firms do not modify their optimal strategies, either in prices or in sustainability characteristics with respect to the unregulated market, so in this context the environmental regulation policy has no effect.

#### 4.2.3. Third Result: Interior Solution

From the last possibility (third case (iii)), corresponding to a relatively higher level of the AC, it is shown that

**Proposition** **3.** *For any*t>0, θ∈[0, 1], *and*$Max\left\{\frac{t}{4\theta },\frac{t}{4\left(1-\theta \right)}\;\right\}\leq \gamma \leq \bar{\gamma }$, *there is a unique equilibrium*$\left(x_{1}^{E3},x_{2}^{E3}\right)$, *given by*

(29) $$\;x_{1}^{E3}=\theta -\frac{t\left(4\theta +1\right)}{4\left(t+\gamma \right)}\;,\;x_{2}^{E3}=\theta +\frac{t\left(5-4\theta \right)}{4\left(t+\gamma \right)}.\;$$

**Proof.**  For  θ∈[0,1] and condition $Max\left\{\frac{t}{4\theta },\frac{t}{4\left(1-\theta \right)}\;\right\}\leq \gamma \leq \bar{\gamma }$, it is verified that $x_{1}^{*}\in \left[0,1\right]$ and $\;x_{2}^{*}\in \left[0,1\right]$, therefore the equilibrium is $\;\left(x_{1}^{E3}=x_{1}^{*},\;x_{2}^{E3}=x_{2}^{*}\right)$. □

##### Comments

CP 3.1. Considering that θ∈[0, 1] into the condition $Max\left\{\frac{t}{4\theta },\frac{t}{4\left(1-\theta \right)}\;\right\}\leq \gamma ,$ it follows that the equilibrium $\left(x_{1}^{E3},x_{2}^{E3}\right)$ is always reached whenever the budget, γ, satisfies the constraint

(30) $$\frac{t}{2}\leq \gamma \leq \bar{\gamma }.\;$$

The environmental regulation will alter the optimal business decisions only if the budget is strictly greater than (t/2). In the opposite case, at least one of the two firms will make identical decisions to the unregulated case (as shown in the previous Section 4.2.1 and Section 4.2.2).

CP 3.2. It is easy to verify that

(31) $$0\leq x_{1}^{E3}\leq \theta \leq x_{2}^{E3}\leq 1.$$

Firm 1 chooses a less sustainable characteristic with respect to the planner’s sustainability benchmark, while firm 2 chooses a more sustainable one.

CP 3.3. Furthermore, the two firms have symmetric strategies (In the standard version the firms are symmetrically located at two sides of the midpoint [30], while in He and Deng, where the social effect of CEA is introduced, the firms are symmetrically distributed on both sides of the mean value of the products purchased by all consumers ), and they are equally distributed on both sides of the characteristic s whose expression is given by

(32) $$s=\frac{x_{1}^{E3}+x{\;}_{2}^{E3}}{2}=\theta +\frac{t\left(\;1-2\;\theta \right)}{2\;\left(t+\gamma \right)}.\;$$

This symmetry leads to singular properties on other elements of the firms, such as the differentiation of products, prices, demands, and benefits.

CP 3.4. The product differentiation denoted by $Z_{R}^{E3}=\left(x_{2}^{E3}-x_{1}^{E3}\right)$ is independent of the planner’s sustainability benchmark θ; however it depends on the budget γ of the AC and the degree of consumers’ environmental sensitivity, t, being

(33) $$Z_{R}^{E3}=\frac{3t}{2\left(t+\gamma \right)}.$$

CP 3.5. The prices $\left(p_{1}^{E3},p_{2}^{E3}\right)$ are equal, and are formulated as

(34) $$\;p_{1}^{E3}=p_{2}^{E3}=t\;Z_{R}^{E3}=\frac{3t^{2}}{2\left(t+\gamma \right)}.$$

CP 3.6. In addition, the demand functions are equal, and given by

(35) $$\;Q_{1}^{E3}=\hat{x}=\frac{1}{2},\;Q_{2}^{E3}=1-\hat{x}=\frac{1}{2}.$$

CP 3.7. Therefore, the firms’ profits are equal, and they are expressed as follows

(36) $$\;B_{1}^{E3}=B_{2}^{E3}=\frac{t}{2}Z^{E3}=\frac{3t^{2}}{4\left(t+\gamma \right)}$$

The environmental regulation does not affect both firms’ demand, since none of them loses buyers compared to the unregulated case. However, it does affect product differentiation and therefore prices and profits. From the previous expressions, it follows that the larger the budget, the greater the competition, and the closer the firms are to the planner’s recommendations.

The following section will examine the optimal strategies of the environmental planner. Given the sequence of decision making, the symmetry point s will be a determining factor in the planner’s behaviour.

### 4.3. Planner’s Optimal Strategies

Considering the firms’ optimal strategies, $\left(x_{1}^{E3},x_{2}^{E3}\right)$, the next step consists in the determination of the optimal strategies of the environmental planner, namely the sustainability benchmark θ, and the budget γ, that minimize social damage per unit spent, Φ(θ,γ) given by Equation (9). Thus, substituting $\left(x_{1}^{E3},x_{2}^{E3}\right)\;$ given by Equation (29) and the result concerning the indifferent consumer $\hat{x}$ given by Equation (35) into Equation (9), the following function is obtained:

(37) $$\Phi_{T}^{E3}\left(\theta ,\gamma ,t\right)=\frac{t^{2}}{16\;\gamma {\left(t+\gamma \right)}^{2}}\left\{9+4{\left(2\theta -1\right)}^{2}\;\right\}.\;$$

Considering first the necessary condition and then the associated sufficient condition, the result found can be summarized as follows:

**Proposition** **4.** *For any*t>0, θ∈[0, 1], *and*$Max\left\{\frac{t}{4\theta },\frac{t}{4\left(1-\theta \right)}\;\right\}\leq \gamma \leq \bar{\gamma }$, *with a private duopoly, the optimal environmental strategies are*:

(38) $$\;\theta^{E3}=\frac{1}{2},\;\gamma^{E3}=\bar{\gamma }\;.\;$$

**Proof.**  Considering $\partial \Phi_{T}^{E}\left(t,\gamma ,c\right)$, given by Equation (37), the necessary conditions are given:

$$\frac{\partial \Phi_{T}^{E}\left(t,\gamma ,\;\theta \right)}{\partial \;\theta }=\frac{\;t^{2}\left(2\;\theta -1\right)}{\gamma {\left(t+\gamma \right)}^{2}}=0$$

$$\frac{\partial \Phi_{T}^{E}\left(t,\gamma ,\;\theta \right)}{\partial \gamma }=\frac{\left(t-\gamma \right)\;t^{2}\left\{\;4{\left(2\;\theta -1\right)}^{2}+9\;\right\}}{16\gamma^{2}{\left(t+\gamma \right)}^{3}}<0$$

and sufficient conditions are:

$$\frac{\partial^{2}\Phi_{T}^{E}\left(t,\gamma ,\theta \right)}{\partial^{\;\theta 2}}=\frac{\;2t^{2}}{\gamma {\left(t+\gamma \right)}^{2}}$$

$$\frac{\partial^{2}\Phi_{T}^{E}\left(t,\gamma ,\theta \right)}{\partial \gamma^{2}}=\frac{\;t^{2}\left(6\gamma^{2}t+4\gamma t+t^{2}\right)\left\{\;4{\left(2\theta -1\right)}^{2}+9\;\right\}}{8\gamma^{2}{\left(t+\gamma \right)}^{4}}$$

$$\frac{\partial^{2}\Phi_{T}^{E}\left(t,\gamma ,\theta \right)}{\partial \;\theta^{2}}=\frac{\partial^{2}\Phi_{T}^{E}\left(t,\gamma ,\theta \right)}{\partial \gamma \;\partial \theta }=-\frac{\;t^{2}\left(2\theta -1\right)\left(t+3\gamma \right)}{2\gamma^{2}{\left(t+\gamma \right)}^{4}},$$

From these equations, it follows we have:

$$\frac{\partial^{2}\Phi_{T}^{E}\left(t,\gamma ,\;\theta \right)}{\partial \theta^{2}}>0,\;\frac{\partial^{2}\Phi_{T}^{E}\left(t,\gamma ,c\right)}{\partial \gamma^{2}}\frac{\partial^{2}\Phi_{T}^{E}\left(t,\gamma ,c\right)}{\partial \;\theta^{2}}-{\left(\frac{\partial^{2}\Phi_{T}^{E}\left(t,\gamma ,c\right)}{\partial \theta^{2}}\right)}^{2}>0,$$

Therefore, the minimum is reached for

$$\theta^{E3}=\frac{1}{2},\;\gamma^{E3}=\bar{\gamma }\;.$$

 □

CP 4.1. The optimal sustainability benchmark $\theta^{E3}$ and the optimum budget $\;\gamma^{E3}$ correspond respectively to the average level of all the sustainability characteristics in the market, and to the highest possible level of the budgets. Now, if the optimal budget $\gamma^{E3}$ seems to be the most appropriate, the optimal characteristic $\theta^{E3}$ is not. In the present model, the sustainability characteristics are labelled in increasing order from 0 to 1, so the planner’s choice of the optimal characteristic is not the most sustainable and may appear a priori to be an inadequate outcome. However, such a decision is not unexpected; it anticipates the choice of symmetric optimal sustainability characteristics by firms. Indeed, in that case, the only way to induce firms to avoid the production of extreme levels of sustainability and reduce product differentiation is to set the average characteristic as the sustainability benchmark supported by a high budget. Finally, the planner promotes an average sustainability characteristic that leads to the less sustainable firm tending to move towards it and thus improve its sustainability, while for symmetric reasons the more sustainable firm will produce a less sustainable characteristic.

CP 4.2. Given the result, the optimal budget restriction $\gamma^{E3}$ is expressed by

(39) $$\bar{\gamma }\geq \frac{t}{2}.$$

CP 4.3. The equilibrium in characteristics $\;(x_{1}^{E3},\;x_{2}^{E3})$ is written as

(40) $$\;x_{1}^{E3}=\frac{1}{2}-\frac{3t}{4\left(t+\bar{\gamma }\right)},\;\;x_{2}^{E3}=\frac{1}{2}+\frac{3t}{4\left(t+\bar{\gamma }\right)}.$$

It is deduced that

(41) $$0\leq x_{1}^{E3}\leq \frac{1}{2}\leq x_{2}^{E3}\leq 1$$

The sustainability characteristic of firm 1, $x_{1}^{E3}$, is increasing, whereas firm 2’s characteristic, $x_{2}^{E3}$, is decreasing with respect to $\bar{\gamma }$. For large values of $\bar{\gamma }$, both firms tend to move towards the centre. Now, with respect to t, the characteristic of firm 1 decreases and that of firm 2 increases. For $t\to 2\;\bar{\gamma }$, the firms move towards the extremes of the market. This shows the opposite effects between the level of environmental sensitivity of consumers t and the budget value $\bar{\gamma }$ assigned to the AC on $(x_{1}^{E3},x_{2}^{E3})\;$.

CP 4.4. The firms are symmetrically located on both sides of the point s given by

(42) $$s=\frac{1}{2}.$$

CP 4.5. Substituting γ by $\bar{\gamma }$ into (33), the product differentiation $Z_{R}^{E3}$ is expressed as

(43) $$\;Z_{R}^{E3}=\frac{3t}{2\left(t+\bar{\gamma }\right)}.$$

**Figure 1:**
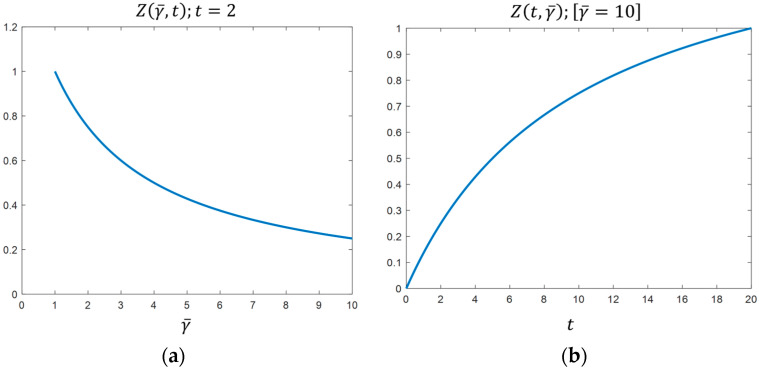
Budget value $\bar{\gamma }$ assigned to the AC on product differentiation.

$Z^{E3}$ is decreasing with respect to $\bar{\gamma }$ and increasing with respect to t. On the one hand, when the budget $\bar{\gamma }$ increases, the firms are approximating, i.e., there is minimum differentiation. On the other hand, when t increases, the firms are reaching maximum differentiation. Similar to the case of optimal sustainability characteristics, there is an opposite effect of consumers’ level of environmental sensitivity t and the budget value $\bar{\gamma }$ assigned to the AC on product differentiation $\;Z^{E3}$. For example, in Figure 1a, $\;Z^{E3}$ is represented as a function of budget,$\;\bar{\gamma }$, for t=2  and in the Figure 1b as a function of t, for $\bar{\gamma }=10$.

The above graphic representations clearly show the opposite effects between t and $\bar{\gamma }$.

CP 4.6. It follows that prices and firms’ profits are reformulated as

(44) $$\;p_{i}^{E3}=\frac{3t^{2}}{2\left(t+\bar{\gamma }\right)},\;B_{i}^{E3}=\frac{3t^{2}}{4\left(t+\bar{\gamma }\right)},\;i=1,2.$$

Prices and profits are also decreasing with $\bar{\gamma }$ and increasing with t. This shows that regulation intensifies price competition and therefore reduces corporate profits, while the degree of environmental sensitivity of consumers produces the opposite result.

CP 4.7. The objective function of the planner is

(45) $$\;\Phi_{T}^{E3}\left(\bar{\gamma },\;t\;\right)=\frac{9\;t^{2}}{16\;\bar{\gamma }{\left(t+\bar{\gamma }\right)}^{2}}.\;$$

**Figure 2:**
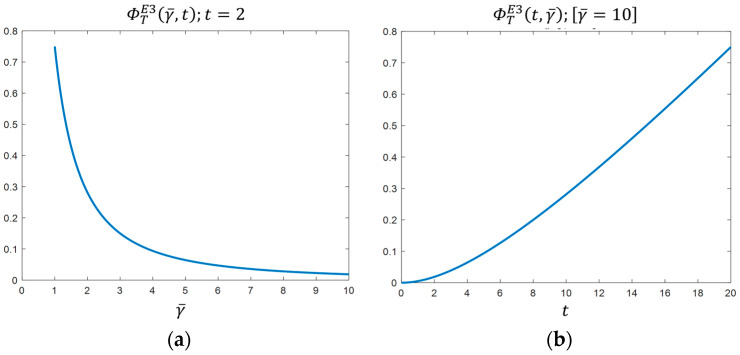
Budget value $\bar{\gamma }$ on the objective function of the planner.

The planner is interested in the budget $\bar{\gamma }$ being maximum and the degree of environmental sensitivity of consumers t  being minimum. In other words, he/she would like consumers to pay more attention to its consumption advice. On the other hand, it has been shown that the firms are interested in the budget being minimum and the degree of environmental sensitivity of consumers being maximum. To reflect the level of environmental sensitivity of consumers t and the budget value $\bar{\gamma }$ on the objective function of the planner, $\;\Phi_{T}^{E3}\left(\bar{\gamma },\;t\;\right)$ is depicted respectively as a function of the budget $\bar{\gamma }$ for t=2 (Figure 2a) and as a function of consumers’ subjective awareness  t for $\bar{\gamma }=10$ (Figure 2b).

Comparing Figure 1a,b respectively with Figure 2a,b, one can clearly see the opposite effects of consumers’ environmental sensitivity level *t* and the budget value $\bar{\gamma }$ on the private duopoly and the planner. There is a rivalry between the objectives of the two parties.

In any case, the results show that the key to environmental issues revolves around the budget $\bar{\gamma }$. The higher the budget, the more effective the regulation will be. The level of intensity of the AC will depend on the budget that the government provides to the planner. Consequently, the efficiency of regulation depends on government policy regarding environmental sustainability. In addition, there will be a positive spillover effect on consumers: as the budget for the AC increases, prices and product differentiation between firms will decrease, and economic competition will intensify.

## 5. Optimal Strategies from a Social Perspective—Public Duopoly

This section studies the model from a social perspective assuming public management of firms. As in the private case, the effects of regulation will be examined. It is considered that the public manager and the “environmental planner” are two independent and autonomous entities. The interaction between the two social agents is analysed as a two-stage sequential game. In Section 5.1, the planner chooses simultaneously the sustainability benchmark  θ and the budget  γ, and in Section 5.2, a public management planner sets the production sustainability characteristics $\;\left(x_{1},x_{2}\right)$.

### 5.1. Maximising Characteristics

The optimal sustainability characteristics of both firms are examined under the public provision approach. Assuming identical prices for both firms$,\;\;p_{1}=p_{2},$ the indifferent consumer is given by

(46) $${\hat{x}}^{S}=\frac{\left(t+\gamma \right)\left(x_{2}+x_{1}\right)}{2t}-\frac{\gamma \theta }{t}\;,\;$$

Substituting $\;{\hat{x}}^{S}$ in the objective function $\;W\left(x_{1},x_{2}\right)$ of the public manager given by (8), the following optimal characteristics are obtained

**Proposition** **5.** *For any*t>0, θ∈[0,1]*and*$\gamma \in \left[0,\bar{\gamma }\right]\;$*the optimal sustainability characteristics are*:

(47) $$\;x_{1}^{S}=\theta -\frac{t\left(4\theta -1\right)}{4\left(t+\gamma \right)}\;,\;\;x{\;}_{2}^{S}=\theta +\frac{t\left(3-4\theta \right)}{4\left(t+\gamma \right)}.$$

**Proof.**  From a social perspective, the planner chooses the characteristics $\;\left(x_{1},x_{2}\right)$ that maximize the social welfare function $\;W\left(x_{1},x_{2}\right)$ given by the Equation (12), substituting $\hat{\;x}$ with ${\hat{x}}^{S}$. The first-order conditions are as follows:

(EQ1) $$\frac{\;\partial W\left(x_{1},x_{2}\right)}{\partial x_{1}}=0\Leftrightarrow \;\left(t+\gamma \right)\left(3x_{1}-x_{2}\right)+2\gamma \theta =0,\;$$

(EQ2) $$\frac{\;\partial W\left(x_{1},x_{2}\right)}{\partial x_{1}}=0\Leftrightarrow \left(t+\gamma \right)\left(3x_{2}-x_{1}\right)-2\gamma \theta -t\left(1-2x_{2}\right)+2\gamma \left(x_{2}-\theta \right)=0$$

Now, clearing $\;x_{2}$ from Equation  (EQ1) and substituting into Equation  (EQ1), the following equation is obtained

$$8\;{\left(t+\gamma \right)}^{2}{\left(x_{1}-\frac{\gamma \theta }{t+\gamma }\;\right)}^{2}-6\left(t+\gamma \right)\left(x_{1}-\frac{\gamma \theta }{t+\gamma }\;\right)t+t^{2}=0.$$

whose solution is given by the expression (47). It is easily verified that $\;x_{1}^{S}\in \left[0,1\right]$, and $x_{2}^{S}\in \left[0,1\right]$. Calculating the Hessian $\;H\left(x_{1}^{S},x_{2}^{S}\right)$, the sufficient condition is verified. □

#### Comments

CP 5.1. Unlike the equilibrium in the characteristics of the private duopoly, here there is no restriction on the budget γ nor on the maximum budget $\bar{\gamma }$. Assuming γ=0, the results are identical to those obtained by the public management in the classic model without environmental regulation [28].

CP 5.2. Comparing this equilibrium to the private duopoly equilibrium $\left(x{\;}_{1}^{E3},\;x{\;}_{2}^{E3}\right)$, it follows that

(48) $$\;x_{1}^{S}=x{\;}_{1}^{E3}+\frac{t}{2\left(t+\gamma \right)},\;x_{2}^{S}=x{\;}_{2}^{E3}-\frac{t}{2\left(t+\gamma \right)}\;.\;$$

from which it is obtained

(49) $$0\leq \;x{\;}_{1}^{E3}\leq \;x{\;}_{1}^{S}\leq x_{2}^{S}\leq x_{2}^{E3}\leq 1.\;$$

The public manager improves the sustainability of the optimal characteristic of firm 1 while worsening that of firm 2.

CP 5.3. From Equation (45) it follows:

(50) $$s=\frac{x_{1}^{E3}+x{\;}_{2}^{E3}}{2}=\frac{x_{1}^{S}+x{\;}_{2}^{S}}{2}\;$$

Thus, as in private duopoly, the two firms are symmetric with respect to the point s.

C.P 5.4. Product differentiation, denoted by $Z_{R}^{S},$ depends only on the budget γ of the AC and the degree of consumer environmental sensitivity, t. From Equation (49), an improvement over the private duopoly follows, which results in

(51) $$\;Z_{R}^{S}=\frac{1}{3}Z_{R}^{E3}=\frac{t}{2\left(t+\gamma \right)}.\;$$

CP 5.5. As usual, in the public duopoly, there is no price competition, regardless of environmental regulation, thus prices are considered fixed and equal

(52) $$p_{1}^{S}=p_{2}^{S}\;$$

CP 5.6. As expected, irrespective of environmental regulation, the demands are again equally distributed.

(53) $$Q_{1}^{S}=Q_{1}^{E3}={\hat{x}}^{S}={\hat{x}}^{E3}=\frac{1}{2},\;Q_{2}^{S}=Q_{2}^{E3}=1-{\hat{x}}^{S}=\frac{1}{2}.$$

CP 5.7. The objective function $W_{S}$ of the public manager, given in Equation (8), is formulated as

(54) $$\;W_{S}\left(\theta ,\;\gamma ,t\right)=K+\frac{t\left[16\gamma \;\theta \left(1-\theta \right)+5t\right]}{16\;\left(t+\gamma \right)}+\frac{t}{3},\;$$

Unlike private duopoly profit functions, here the objective function of the public manager $W_{S}$ depends not only on the budget γ but also on the benchmark characteristic θ. Regardless of the value of θ, when γ=0 (without AC), the above-mentioned result is obtained (see comment CP 5.1). Again, the budget γ is crucial to incentivising the change in the sustainability level of its output.

Next, the first stage of the game is analysed, where the environmental planner simultaneously chooses its two strategic variables: the sustainability benchmark θ and the budget γ.

### 5.2. Optimal Strategies of the Environmental Planner 

By substituting $\;\left(x_{1}^{S},x_{2}^{S}\right)$ and the indifferent consumer $\;{\hat{x}}^{S}$ in the given Equation (10), the damage function is reformulated as follows:

(55) $$\;\Phi_{T}^{S}\left(\theta ,\gamma ,t\right)=\frac{t^{2}}{16\gamma {\left(t+\gamma \right)}^{2}}\left\{\;1+4\;{\left(2\theta -1\right)}^{2}\right\}.\;$$

The expression of the objective function of the environmental planner is formally similar to that obtained in the case of privately managed firms. Specifically, the relationship between both scenarios is given by

(56) $$\;\Phi_{T}^{S}\left(\theta ,\gamma ,t\right)=\Phi_{T}^{E3}\left(\theta ,\gamma ,t\right)-\frac{t^{2}}{2\gamma {\left(t+\gamma \right)}^{2}}.\;$$

Therefore, the planner’s optimal instruments are identical to those of the private duopoly case as formalised in the following proposition.

**Proposition** **6.** *For any*t>0, θ∈[0, 1], *and*$0\leq \gamma \leq \bar{\gamma }$, *with a public duopoly, the optimal environmental strategies are*:

(57) $$\;\theta^{S}=\frac{1}{2},\;\gamma^{S}=\bar{\gamma }\;.\;$$

**Proof.**  Considering the relationship between the objective functions considered in Section 4 and in Section 6 given by Equation (56), this proof is identical to fourth Proof. □

#### Comments

CP 6.1. Due to the similarity of the firms’ symmetry between the public and private scenario (see Proposition 5 and comment CP 5.3.), the optimal strategies of the environmental planner and their justifications are identical to the private duopoly scenario (see Proposition 3 and comment CP 3.1.).

CP 6.2. Now, regarding other results that can be deduced from this proposition, such as the budget restriction, the characteristics of the firms, product differentiation, prices, and demand, all are similar to the comments in Proposition 5 (specifically see CP 5.1., CP5.2., CP 5.3., CP 5.4., and CP 5.5.) with the difference that θ is replaced by (1/2) and γ by $\bar{\gamma }$.

CP 6.3. Substituting θ and γ respectively by $\theta^{E3}=\frac{1}{2}$, and $\gamma^{E3}=$ $\bar{\gamma }$ into Equation (54), the objective function $W_{S}\left(\theta ,\;\gamma ,\;t\right)$ of the public manager is formulated as follows

(58) $$\;W_{S}\left(\;\bar{\gamma },t\right)=K+\frac{t\left[4\bar{\gamma }\;+5t\right]}{16\;\left(t+\bar{\gamma }\right)}+\frac{t}{3}.$$

Similar to the profit functions of the private duopoly, $W_{S}\left(\;\bar{\gamma },\;t\right)$ decreases with respect to $\bar{\gamma }$ and increases with respect to t, and again the effects of $\left(\bar{\gamma }\right)$ and (t) are opposite.

CP 6.4. The objective function of the environmental planner

(59) $$\Phi_{T}^{S}\left(\bar{\gamma },t\;\right)=\frac{\;t^{2}}{16\;\bar{\gamma }{\left(t+\bar{\gamma }\right)}^{2}}.$$

Compared to $\Phi_{T}^{E3}\left(\bar{\gamma },t\;\right)$, it is obtained

(60) $$\;\Phi_{T}^{S}\left(\bar{\gamma },t\;\right)=\frac{1}{9}\Phi_{T}^{E3}\left(\bar{\gamma },t\;\right).\;$$

Therefore, the properties and the graphical representation of the two functions are similar.

Given the results obtained, it emerges that regardless of the type of market considered, whether a private or public duopoly, there is a clear antagonism between the economic forces (firms or public management) and the promoter of environmental regulation (planner). Moreover, the antagonism is even stronger in a private duopoly scenario. The comparison between the private duopoly and public duopoly approaches, with and without environmental regulation, will be further developed below, where the differences and similarities will be clearly explained.

## 6. Comparisons

In the standard unregulated model, Tirole shows that the optimal characteristics of a private duopoly are different from those of a public duopoly, concluding that the market outcome leads, socially, to too large product differentiation [29]. The firms are in the first and third quartile, and differentiation is reduced by half compared to the private duopoly outcome. In the present model, considering environmental regulation, the public duopoly still performs better in terms of product differentiation than the private duopoly, the improvement being one third. In the case of environmental regulation, the differentiation results depend on the budget level $\bar{\gamma }$ and consumer sensitivity t.

In order to clearly distinguish and compare the degree of product differentiation between the four scenarios mentioned above, we denote as:

1. $Z_{NR}^{E}$ the differentiation of a private duopoly without environmental regulation,

(61) $$\;Z_{NR}^{E}=1;$$

2. $Z_{NR}^{S}$ the differentiation of a public duopoly without environmental regulation,

(62) $$\;Z_{NR}^{S}=\frac{1}{2};$$

3. $Z_{R}^{E3}\left(\;\bar{\gamma },t\right)$ the differentiation of a private duopoly with environmental regulation, whose expression is given by Equation (43);
4. $Z_{R}^{S}\left(\;\bar{\gamma },t\right)$ the differentiation of a public duopoly with environmental regulation, whose expression is given by Equation (51) substituting γ by $\bar{\gamma }$.

Comparing the degree of product differentiation of the distinct scenarios 1, 2, 3, and 4, the following results are obtained.

**Proposition** **7.** *A public duopoly with environmental regulation leads to less product differentiation*.

**Proof.** 

i. The difference between the product differentiation of the public duopoly with environmental regulation $Z_{R}^{S}\left(\;\bar{\gamma },t\right)$ and $Z_{R}^{E3}\left(\;\bar{\gamma },t\right)\;$ is given by
  $$\;I_{1}=Z_{R}^{S}\left(\;\bar{\gamma },t\right)-Z_{R}^{E3}\left(\;\bar{\gamma },t\right)=\frac{-2\;t}{\left(t+\bar{\gamma }\;\right)}<0.$$

ii. The difference between the product differentiation of the public duopoly with environmental regulation $Z_{R}^{S}\left(\;\bar{\gamma },t\right)$ and $Z_{NR}^{S}$ is given by
  $$\;I_{2}=Z_{R}^{S}\left(\;\bar{\gamma },t\right)-Z_{NR}^{S}=\frac{-\bar{\gamma }}{2\left(t+\bar{\gamma }\;\right)}<0.$$

iii. The difference between the product differentiation of the public duopoly with environmental regulation $Z_{R}^{S}\left(\;\bar{\gamma },t\right)$ and $Z_{NR}^{E}$ is given by
  $$\;I_{3}=Z_{R}^{S}\left(\;\bar{\gamma },t\right)-Z_{NR}^{E}=\frac{-\left(t+2\bar{\gamma }\;\right)}{2\left(t+\bar{\gamma }\;\right)}<0.$$

From Equations (59)–(61) it is easy to deduce that

$$\;Z_{R}^{S}\left(\;\bar{\gamma },t\right)<Min\left\{Z_{R}^{E3}\left(\;\bar{\gamma },t\right),\;Z_{NR}^{S},\;Z_{NR}^{E}\left(\;\bar{\gamma },t\right)\right\}$$

Public duopoly with environmental regulation leads to the least differentiation. □

Assuming that the minimum differentiation is obtained with a public duopoly in an environmental regulation scenario and that the maximum differentiation is obtained with the private duopoly without regulation, the intermediate cases are examined below. Specifically, the private duopoly with environmental regulation is compared to the public duopoly without environmental regulation. The purpose of such comparisons is to highlight the level of efficiency of environmental regulation campaigns with a private duopoly. While in the public duopoly environmental regulation is always the most efficient scenario, in the case of private duopoly this is not the case, as shown in the following proposition.

**Proposition** **8.**

(*i*) *For*$\left(t/2\right)\leq \bar{\gamma }\leq 2t$*and*t>0, *the product differentiation in the private duopoly with environmental regulation is greater than or equal to that of the public duopoly without regulation*$\left(Z_{R}^{E3}\left(\;\bar{\gamma },t\right)\geq Z_{NR}^{S}\right)$.

(*ii*) *For*$\bar{\gamma }\geq 2t$*and*t>0, *the product differentiation in the private duopoly with environmental regulation is less than or equal to that of the public duopoly without regulation*$\left(Z_{R}^{E3}\left(\;\bar{\gamma },t\right)\leq Z_{NR}^{S}\right)$.

**Proof.**  As shown in Proposition 4 (CP 4.2.), for $\bar{\gamma }\geq \left(t/2\right)$, there is an equilibrium in sustainability characteristics with a private duopoly in an environmental regulation context. The difference between the differentiation in the public duopoly with environmental regulation and the public duopoly without regulation in equilibrium is given by

(63) $$\;I_{4}=Z_{R}^{E3}\left(\;\bar{\gamma },t\right)-Z_{NR}^{S}=\frac{2t-\bar{\gamma }}{2\left(t+\bar{\gamma }\;\right)}.$$

From Equation (63), it follows that

- If $\left(t/2\right)\leq \bar{\gamma }\leq 2t\;\Rightarrow \;Z_{R}^{E3}\left(\;\bar{\gamma },t\right)\geq Z_{NR}^{S}$

- If $2t\leq \bar{\gamma }\;\Rightarrow \;Z_{R}^{E3}\left(\;\bar{\gamma },t\right)\leq Z_{NR}^{S}$. □

A private duopoly with environmental regulation is always a better scenario than one without environmental regulation. However, this is not always the case compared to a public duopoly without environmental regulation. It all depends on the level of the budget $\bar{\gamma }$ with respect to the degree of environmental sensitivity of consumers, t. In Proposition 8, a budget threshold equal to 2t was determined, below which a public duopoly without environmental regulation would be more efficient. Therefore, it is essential that the budget level $\bar{\gamma }$ that is assigned to campaigns be greater than 2t to induce the public or private duopoly to adopt more sustainable behaviour.

## 7. Conclusions

That awareness campaigns can help change citizens’ consumption behaviour to some extent is certain. However, in order to bring about any change in consumer behaviour, what needs to be done? The question is pertinent and important because our decisions to consume and buy are very much considered in determining what is produced and will therefore affect companies’ market shares and market performance. This work has contributed to shedding light on this issue. To do so, we have developed a simple model in which an environmental authority proposes a sustainability feature that it promotes with an awareness campaign. Some simplifying assumptions have been made to make the analysis tractable, as there is a full impact of the AC on consumers that is reflected in their preferences and regulatory costs are zero. The use of this non-punitive scenario has allowed us to show that preventive regulatory measures can induce firms to lean towards the production orientations of the environmental authority. However, although firms’ responses to regulatory measures are in line with the regulator’s directives, the level of the adjustment depends on the type of duopoly considered: private or public. The two scenarios have provided the following results.

The first scenario is a market with a private duopoly. The optimal strategies of the firms depend only on the two elements of consumers’ environmental awareness, i.e., on the subjective component of consumers’ environmental awareness and on the ACs. However, their effects are opposite in price competition and product differentiation just as in the work of He and Deng [14]. The difference in product environmental quality between firms, prices, and profits are increasing with respect to the subjective component of environmental awareness and decreasing with respect to ACs. This rivalry between the two factors of environmental awareness gives more meaning to the role of the regulator. The subjective element is more often irrational, determined by impulse, emotions, and habits and therefore very complex to manage. Meanwhile, policies can influence consumer behaviour; they have the means, but paradoxically everything depends on these means. The regulator’s optimal characteristic corresponds to the average of the characteristics and the optimal budget corresponds to the maximum budget available to the regulator. In other words, the regulator’s best policy is to invest its maximum budget in the AC. The larger the budget, the larger the AC and the smaller the deviations of the companies from the regulator’s proposal. On the other hand, the strength of the AC helps to intensify competition in characteristics and prices among companies. However, this result is feasible only if the subjective component of environmental awareness is twice lower than the regulatory component, i.e., the AC. Therefore, the public authorities must have a minimum of means in their policies to pretend to achieve a minimum environmental regulation of the private duopoly.

Now, considering the second scenario, i.e., the market with a public duopoly, the results are similar in several formal aspects to those of the first scenario. The optimal strategies of the firms depend solely on the two factors of consumers’ environmental awareness. Their effects are equally opposite. However, competition in terms of features and prices is more intense, and the profits of firms are lower.

This result is rather due to the public framework. In any case, the antagonism of the effects of the two components of environmental awareness is still present. Therefore, the intervention of an environmental authority remains important. The regulator’s optimal strategies are identical to those of the first scenario, with a difference that is not unimportant. This result is not constrained, i.e., the regulator does not need to have a minimum budgetary capacity to improve the behaviour of the companies. The market is improved by public management of the companies alone. However, maximum investment is still important. The same motto as before applies: the larger the budget, the larger the AC, and the greater the commitment of the companies to the regulator.

Based on all the above observations, we would like to comment on the results that show some limitations of the Hotelling model [30,31] and highlight several extensions that are worth considering. The optimal characteristic proposed by the regulator corresponds to the average of all characteristics. This decision is closely related to the optimal decisions of firms that are symmetric. Indeed, the symmetry of the firms distorts the regulator’s behaviour by forcing it in a certain way to take an average position between the two firms. The equilibrium in the standard Hotelling model is ensured with quadratic transport costs resulting in the symmetric behaviour of the firms [26]. That resulting symmetry is a constraint on applying Hotelling in several issues, and specifically regulation [32]. An extension of our model that can further clarify the behaviour of the regulator is a consideration of different production costs for the firms, which will obviously complicate the resolution. Another extension is to introduce other intervention mechanisms, such as the imposition of fines/subsidies in the standard Hotelling model, since the tax/subsidy incentivizes producers of green and brown goods to reduce pollution. Moreover, considering that environmental authorities are increasingly aware of the importance of coordinating their actions to achieve a sustainable production system, it would be desirable to combine awareness campaigns and taxes or subsidies in the same model. This will make it possible to measure the degree of complementarity between the two instruments. Another interesting aspect to investigate would be to consider the impact of AC on only a proportion of consumers as a whole. Consumer awareness is a complex task with results that are difficult to predict. On the other hand, the subject of the model as well as the proposed extensions can be analysed, in vertical differentiation markets, in a context of price discrimination or competition in quantities.

This article contributes to the literature on environmental policy and industrial management of firms. It provides a framework for how planners should determine the appropriate sustainability benchmark and carry out the AC that supports it. It also determines the most appropriate business scenario to achieve the most efficient results.

## Data Availability

Not applicable.
